# Preparation of Photoactive Transition-Metal Layered Double Hydroxides (LDH) to Replace Dye-Sensitized Materials in Solar Cells

**DOI:** 10.3390/ma13194384

**Published:** 2020-10-01

**Authors:** Sajid Naseem, Bianca R. Gevers, Frederick J. W. J. Labuschagné, Andreas Leuteritz

**Affiliations:** 1 Processing Department, Leibniz Institute of Polymer Research Dresden, 01069 Dresden, Germany; 2 Institute of Materials Science, Technical University (TU) Dresden, 01069 Dresden, Germany; 3 Department of Chemical Engineering, Institute of Applied Materials, University of Pretoria, Pretoria 0002, South Africa; bianca.gevers@tuks.co.za (B.R.G.); johan.labuschagne@up.ac.za (F.J.W.J.L.)

**Keywords:** layered double hydroxide solar cell (LDHSC), photoactive material, UV-Vis absorption, dye sensitized solar cell (DSSC), photoactive layered double hydroxide (LDH), transition metal modification, optical bandgap analysis, renewable energy, photovoltaic device design, iron (Fe) modified MgFeAl LDH

## Abstract

This work highlights the use of Fe-modified MgAl-layered double hydroxides (LDHs) to replace dye and semiconductor complexes in dye-sensitized solar cells (DSSCs), forming a layered double hydroxide solar cell (LDHSC). For this purpose, a MgAl-LDH and a Fe-modified MgAl LDH were prepared. X-ray diffraction spectroscopy (XRD), scanning electron microscopy (SEM), and energy-dispersive X-ray (EDX) spectroscopy were used to analyze the structural properties, morphology, and success of the Fe-modification of the synthesized LDHs. Ultraviolet-visible (UV-Vis) absorption spectroscopy was used to analyze the photoactive behavior of these LDHs and compare it to that of TiO_2_ and dye-sensitized TiO_2_. Current-voltage (I–V) solar simulation was used to determine the fill factor (FF), open circuit voltage (V_OC_), short circuit current (I_SC_), and efficiency of the LDHSCs. It was shown that the MgFeAl-LDH can act as a simultaneous photoabsorber and charge separator, effectively replacing the dye and semiconductor complex in DSSCs and yielding an efficiency of 1.56%.

## 1. Introduction

With increasing environmental changes because of the wide use of fossil fuels, research into alternative sustainable energy resources is becoming ever more important. Sunlight is an abundantly available energy source that can be harnessed for renewable energy generation. Over the past couple of decades, research into materials suitable for the harvesting of solar light has been on the rise to overcome the pollution problems created by our society [1]. A multitude of approaches has been considered to try to overcome these problems, and the use of solar cells to harvest widely available sunlight and turn it into electricity is one that has shown very good results [2,3]. A large number of different types of solar cells have been developed over the years and research is continuing to find better functioning cells and cells that make use of new materials developed specifically with the aim to be easily manufacturable, sustainable, and cost-effective [4]. Efficiency and final cost are two key factors to consider in the design of new types of solar cells or materials for them. Silicon solar cells are the oldest and most widely used solar cells and have been forerunners in the field, achieving the highest efficiencies but coming with the drawback of high cost and difficult preparation [5,6]. Some low cost alternatives for silicon solar cells exist, dye-sensitized solar cells (DSSCs) being one of them. However, DSSCs have only achieved low efficiencies compared to silicon solar cells [7,8,9].

Since their inception in 1991 by O’Regan and Grätzel [8], DSSCs have received a lot of research attention to try to improve their performance. The improvements attempted have encompassed all parts of the solar cell: the dyes used, the anode material (originally TiO_2_), the counter electrode, and the electrolyte. In DSSCs, light absorption leads to photoexcitation of the dye, subsequent electron injection into the conduction band of the (typically) TiO_2_ anode, and regeneration of the oxidized dye by accepting an electron from the redox system which itself is reduced on the Pt counter electrode [8,10,11,12]. Problems frequently encountered in DSSCs are the cost, long-term stability of the dyes used, and the evaporation of solvents from and leakage of the electrolyte. Some of the problems associated with the electrolyte have been attempted to be solved by using gel, quasi-solid-state, or ionic liquids. A large range of dyes have also been tested for the sake of improvements in oxidation stability or for matching the dye appropriately to the electrolyte [13].

In DSSCs wide bandgap semiconductors are typically used to facilitate appropriate injection of separated charges into the conduction band of these materials [13]. The semiconductor applied to the working electrode is thus typically only active in the UV spectrum and while it can absorb light in this range and separate charges, its main function is frequently to act as a support structure for the dye with high surface area and to effectively transport the charges. Some research has been conducted on the suitability of layered double hydroxides (LDHs) to replace commonly used ZnO or TiO_2_ as wide bandgap semiconductors.

LDHs are anionic clays with a formula of [M_1−x_^2+^M_x_^3+^(OH)_2_]^x+^·[(A^n−^)_x/n_ yH_2_O]^x^, where M^2+^, M^3+^, and A^n−^ are divalent metal cations, trivalent metal cations, and interlayer anions respectively [14]. The study of LDHs has increased widely in different applications because of their wide range of applications and tunable properties [15]. LDHs could also be potential materials for photovoltaic applications because of their promising tailorability with respect to light absorption [16]. Especially Zn- and Ti-based LDHs are good UV-absorbers [17]. For application in DSSCs, LDHs have been used as mixed metal oxides (MMOs) bonded or sintered to ITO on their own or deposited on other wideband semiconductors such as TiO_2_ [18]. They have also found application in quasi-solid-state electrolytes [18,19,20,21,22]. However, currently, very few studies exist that examine the use of LDHs and their MMO or layered double oxide (LDO) derivatives in DSSCs.

Results obtained using liquid electrolytes and containing LDH-MMOs/LDOs as electron conducting materials have shown efficiencies of up to 5.68% (TiO_2_@NiAl-LDO) [23]. Most frequently however, reported results achieve up to 1% efficiency or less, even with changing frequently studied parameters, such as the influence of calcination temperature [24,25] and different dyes [26]. The higher efficiencies reported include the use of a ZnSn-MMO sensitized with D205 (1.28% efficiency) [25], ZnTi-MMO sensitized with C101 (1.57% efficiency) [26] and Zn_73_Al_27_O sensitized with N719 (1.02% efficiency) [27]. The possibility to change the photoanode material, the dye, and the electrolyte, makes it possible to alter and improve the design and efficiency of DSSCs. For this purpose some researchers have exchanged the dye with CdS quantum dots to increase light absorption (3.92% efficiency reached) [28], some have exfoliated and sensitized LDHs with anthraquinone sulfonate (0.2% efficiency reached) [29], and others have come up with different designs which only loosely resemble the DSSC concept [30], and some used LDHs as part of solid electrolytes (8.4% efficiency reached) [21] or combined the LDH as a p-type/n-type heterojunction with polythiophene (0.0032% efficiency reached) [31].

One of the tailoring options we have found to remain unexplored in solar cells using liquid electrolytes is the use of photoactive LDHs as a combined photoabsorber and separated charge generator and conductor. In previous work [16], we have shown that it is possible to modify and tailor the absorbance of simple MgAl-LDHs based on the transition metals (Fe, Co, Ni, Cu, and Zn) used. Different synthesis methods for these transition-metal modified MgAl-LDHs were considered (co-precipitation and urea hydrolysis). It was shown that the effect of the synthesis method significantly alters the morphology and some of the properties of the LDHs, yielding nanostructured materials for the co-precipitated LDHs [32] and large, thin platelets for the urea hydrolysis synthesized materials [33]. Fe-modified MgAl-LDH synthesized using urea hydrolysis, hereby showed the best and strongest absorbance in the MgFeAl-LDH.

In this work, it was thus explored whether such a UV-Vis photoactive MgFeAl-LDH could replace the need for dye-sensitization in liquid electrolyte solar cells. For this purpose, four cells were prepared:

1. A first reference cell containing only MgAl-LDH to determine whether this material on its own could function as a photoabsorber (SCN1).
2. A second reference cell containing plain MgAl-LDH sensitized with dye (Coumarin 153) (SCN2).
3. A third reference cell containing dye-sensitized (Coumarin 153) TiO_2_ (SCN3).
4. A cell containing 5 mol% Fe modified MgFeAl-LDH which has been shown to absorb MgFeAl-LDH (SCN4).

The cells prepared will be referred to as LDHSCs (layered double hydroxide solar cells) in the text.

## 2. Materials and Methods

### 2.1. Materials

Al(NO_3_)_3_·9H_2_O, Fe(NO_3_)_3_·9H_2_O, and Mg(NO_3_)_2_·6H_2_O were purchased from ABCR GmbH (Karlsruhe, Germany). Urea, absolute ethanol, EL-HPE high-performance electrolyte, Coumarin 153, and indium tin oxide (ITO) coated square glass slide with surface resistivity of 8–12 Ω/sq were purchased from Sigma Aldrich. TiO_2_ (SSA: 30 m^2^/g, APS: 30–40 nm, purity: 99%) was purchased from Nanostructured & Amorphous Materials Inc. (Houston, TX, USA). Chemically pure (CP) or analytical grade (AR) reactants were used for all experiments without treatment. Distilled water was used in the LDH synthesis.

### 2.2. Synthesis of MgAl- and MgFeAl-LDHs

MgAl- and MgFeAl–LDHs were synthesized using urea hydrolysis as described in previous work [33]. A MII+: MIII+ molar ratio of 2:1 was used in the synthesis. Fe was substituted as Fe/(Al+Fe) = 0.05 (all on a molar basis). Solutions of Mg(NO_3_)_2_·6H_2_O, Al(NO_3_)_3_·9H_2_O and Fe(NO_3_)_3_·9H_2_O salts were prepared in distilled water and mixed well in a round bottom flask. The solution was heated to 100 °C, and stirred at this temperature for 48 h. After reaction, the mixture was cooled to room temperature, filtered, and thoroughly washed with distilled water. The filtered material was dried in an oven at 70 °C for 24 h.

### 2.3. Preparation of the Solar Cells

**Figure 1:**
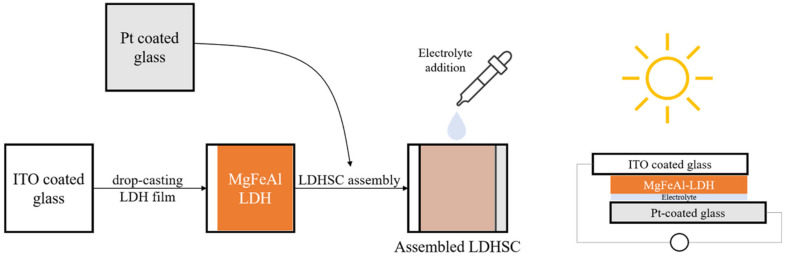
Schematic representation of the preparation of a layered double hydroxide solar cell (LDHSC).

The simplest preparation possible was chosen for the cells. While calcination is a frequent tool in the preparation of solar cells in order to reduce contact resistance between the semiconductor and the conductive material it is deposited on, calcination of the material onto the substrate was excluded in this work in order to retain the photo-response of the MgFeAl–LDH in the UV-Vis region as previously identified [16] and to exclude any possible changes in the material characteristics resulting from calcination. Two types of cells were prepared: dye-sensitized cells and cells making use only of the photoabsorptive capacity of the LDHs. For all four cells, 5 mg of either TiO_2_ or LDH was suspended in 5 mL of absolute ethanol and ground to a paste in a mortar and pestle. The paste was then drop-cast onto ITO coated glass. This paste was allowed to dry fully prior to any additional steps. For the dye-sensitized cells, 0.0096 mL of Coumarin 153 was subsequently dropped onto the material after drying. The prepared photo-anodes were then combined with a Pt-coated ITO glass counter electrode. This electrode was obtained by sputter coating glass with a thin layer of Pt with a SCD 500 sputter coater from Baltec. Finally, 0.0192 mL high-performance electrolyte was injected between the two electrodes to complete the cell. The actual amounts of MgAl–LDH deposited onto the surface of ITO glass were 2.10 mg for MgAl–LDH, 2.17 mg for MgAl–LDH sensitized with dye, 2.15 mg for TiO_2_, and 2.21 mg for MgFeAl–LDH, thus achieving close comparability in material deposition. The cells will be referred to as SCN1, SCN2, SCN3, and SCN4, respectively. The structure and detailed composition of the cells are shown in Table 1, while Figure 1 shows a schematic of the LDHSC preparation procedure utilized.

### 2.4. Characterization Methods

X-ray diffraction spectroscopy (XRD) was performed on a Panalytical X’Pert PRO X-ray diffractometer (Malvern Panalytical, Malvern, UK) in θ–θ configuration, equipped with a Fe-filtered Co-Kα radiation (1.789 Å) and with an X’Celerator detector and variable divergence- and fixed receiving slits. Samples were prepared according to the standardized Panalytical backloading system, which provides nearly random distribution of the particles. The data were collected in the angular range 5° ≤ 2θ ≤ 80° with a step size 0.008° 2 θ and a 13 s scan step time. The phases were identified using X’Pert Highscore plus software. Scanning electron microscopy (SEM) was done using a Zeiss Ultra Plus (Carl Zeiss Microscopy GmbH, Jena, Germany) at 3.00 keV and 6.00 keV for LDH and TiO_2_ respectively. Energy dispersive X-ray spectroscopy (EDX) was done using a QUANTAX FlatQUAD from Bruker Nano GmbH (Berlin, Germany) at 6.00 keV. SEM and EDX samples were prepared in a suspension of ethanol and dropped onto a silicon wafer. The samples were carbon-coated before analysis. UV-Vis absorption spectra of all the samples were obtained using a Lambda 800 from Perkin Elmer (Hamburg, Germany). Bandgap values of all the samples were determined using the absorption spectrum fitting (ASF) method [16,34]. The pellets were prepared by grinding a mixture of KBr and the LDHs or TiO_2_ (70% KBr and 30% LDH, dye sensitized TiO_2_ and TiO_2_) for 60 s. The powder was then pressed into pellets using a pellet press with a pressure of 8 t that was applied for 2 min. The final pellet weighed 300 mg and was 1-mm thick. Pellets of the dye sensitized (TiO_2_ and MgAl) were prepared in the same way. The dye-sensitized powder was obtained in the same way as used in the cell reparation procedure but before injecting the electrolyte. The current-voltage (I–V) curves were obtained using an I–V tester (Jmida SCT-110, JmidaTechnology, Richmond, TX, USA) under simulated AM 1.5 sunlight with an output power of 100 mW/cm^2^ using an I-V solar simulator (Trisol 300 mm solar Simulator, OAI, San Jose, CA, USA) as the light source. The active area of solar cells was about 0.125 cm^2^.

## 3. Results and Discussion

**Figure 2:**
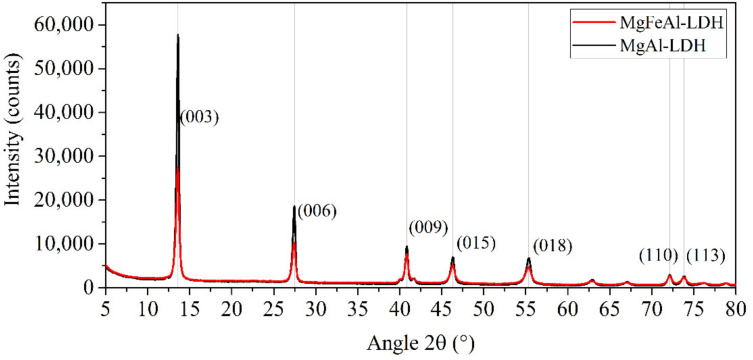
XRD patterns of the MgAl- and MgFeAl–LDH.

**Figure 3:**
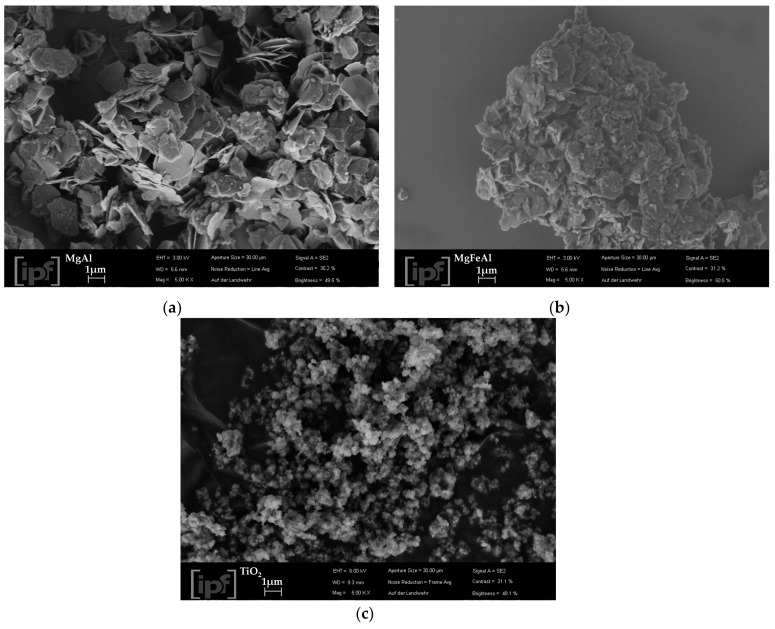
SEM micrographs of the synthesized LDHs (**a**) MgAl-LDH; (**b**) MgFeAl-LDH and (**c**) TiO_2_.

Figure 2 shows the XRD patterns of MgAl- and MgFeAl-LDH. Narrow, high-intensity reflections could be observed corresponding to the (003), (006), and (009) reflections of carbonate intercalated MgAl-LDH in the R$\bar{3}$m space group as described in [32] with high crystallinity and order. The crystallinity of the MgFeAl-LDH was observed to be lower than that of MgAl-LDH, which is expected to be a result of the substitution of Fe causing the formation of small amounts of amorphous material that hinders the crystal growth of the LDH and in turn reduce the crystallinity of the material, as previously observed [34,35], and cause the formation of smaller platelets. Overall, good crystallinity and no appreciable amounts of impurity phases being visible indicated the achievement of Fe-substitution as desired [33]. The XRD observations could be corroborated to the SEM micrographs of the materials showing smaller platelets, more particulate matter and less well-defined platelets for MgFeAl-LDH (Figure 3).

Since these LDHs were prepared using urea-hydrolysis, the platelets were expected to be thin, large, and well-crystallized [36] in comparison to materials formed through co-precipitation. MgAl-LDH better portrayed this hexagonal platelet structure. The well-crystallized nature of these materials is a function of their synthesis procedure, where urea is slowly dissolved in the reaction mixture and leads to a constant low degree of supersaturation [31,35]. The small amounts of impurity-type phases visible, sticking on the Fe-modified LDH platelets were not picked up by XRD as impurities. These phases would thus either need to be crystalline phases present at less than 2% (due to the XRD crystalline phase detection limit) or be of amorphous type. Likely such impurity phases would be oxides or hydroxides of the metals used. Because of no large amorphous bulges being visible on the XRD patterns, only small amounts of an amorphous impurity phase could have been present. Figure 3 also shows the SEM micrograph obtained for TiO_2_, which was confirmed to be a nano-sized material, as specified by the manufacturer.

**Figure 4:**
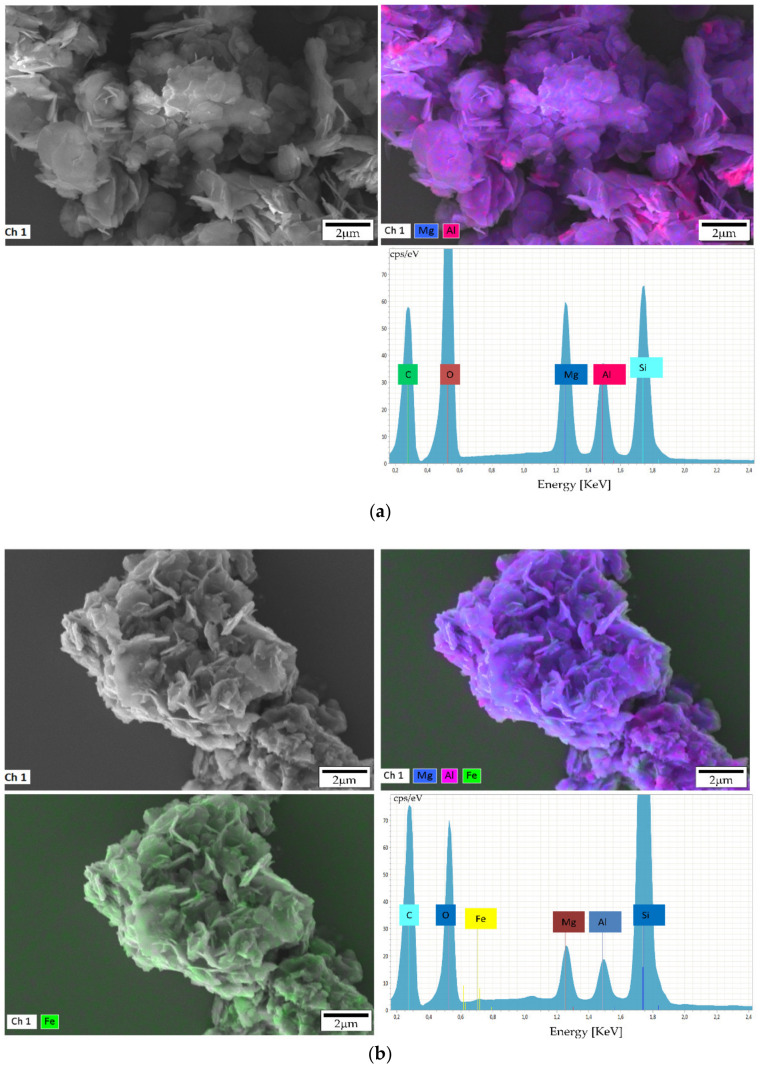
EDX analysis of synthesized LDHs (**a**) MgAl-LDH and (**b**) MgFeAl-LDH.

EDX analysis (Figure 4) showed that Fe was well-dispersed in the MgFeAl-LDH, although some accumulation of the transition metal was visible, as well as some accumulation of Al and Mg. It is expected that brightly colored spots (indicating a greater accumulation of the metal) are the result of some amorphous material or very small crystalline phase formed, as previously discussed. It could be shown that a good distribution of the Fe in the LDHs layers was achieved with only very small amounts of residue material on the LDH platelets visible.

**Figure 5:**
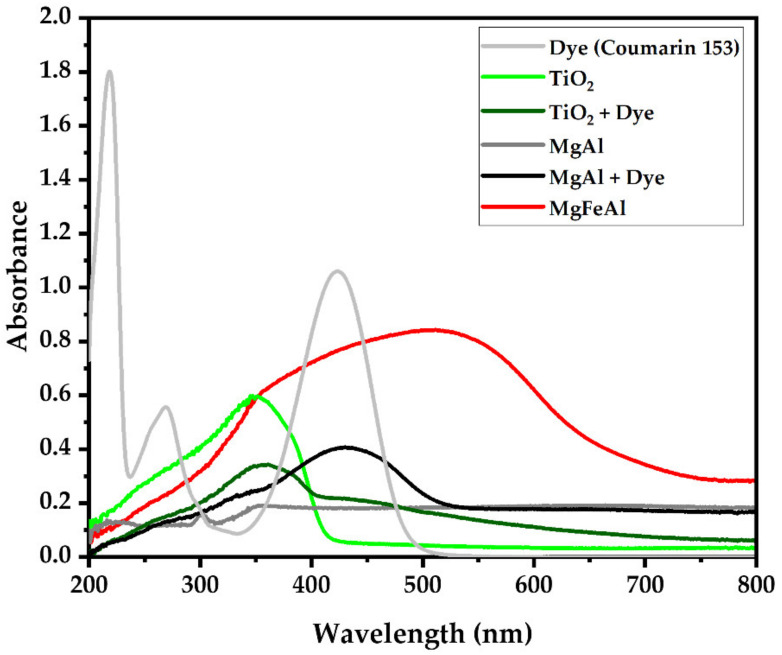
UV-Vis absorption spectra of dye, TiO_2_, TiO_2_+dye, MgAl, MgAl+dye, and MgFeAl.

Figure 5 shows the UV-Vis absorption spectra of dye (Coumarin 153), TiO_2_, TiO_2_ sensitized with Coumarin 153, MgAl-LDH, MgAl-LDH sensitized with Coumarin 153 and MgFeAl-LDH. MgAl-LDH only showed a small absorption band in the UV-region. MgFeAl-LDH exhibited a strong absorption band spanning the UV-Vis region. UV-Vis absorbance was greatly enhanced by the Fe-substitution in comparison to MgAl-LDH. Comparison of the UV-Vis results of the LDHs and the TiO_2_ (photosensitized and plain) showed that the Fe-substituted LDH absorbed considerably more light in the visible region. Coumarin 153 sensitization led to an increase in absorption between approximately 350 nm and 500 nm, as expected because of the absorption range of the dye itself. All LDHs showed a “background” absorbance, as has been previously noted [16].

**Figure 6:**
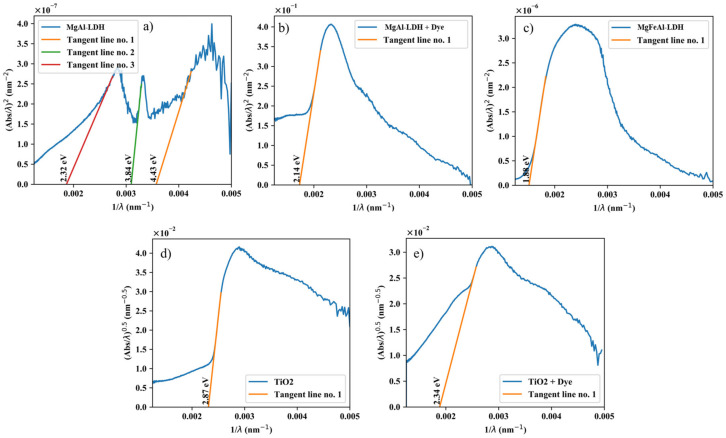
Bandgap determination of (**a**) MgAl-LDH (2.32 eV, 3.84 eV and 4.43 eV); (**b**) MgAl-LDH+dye (2.14 eV); (**c**) MgFeAl-LDH (1.88 eV); (**d**) TiO_2_ (2.87 eV); and (**e**) TiO_2_+dye (2.34 eV) using the ASF method.

The bandgap of all materials was determined using the ASF method as described by Ghobadi et al. [37], the results of which are shown in Figure 6.

**Figure 7:**
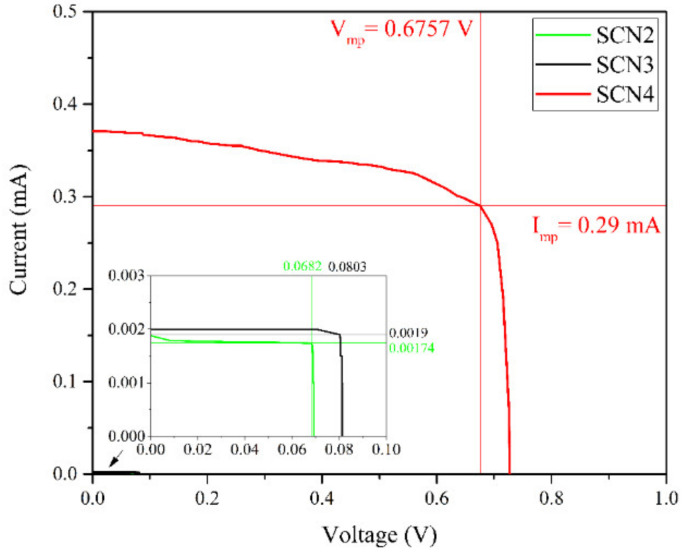
Current (I)-voltage (V) characteristics curves of LDH solar cells (LDHSC)**.**

For the use of the ASF method, it was assumed that the LDHs act as direct bandgap semiconductors. LDHs can act as direct and indirect bandgap semiconductors [38], especially with transition-metal modification. However, using indirect transition types, the bandgaps obtained could not be consolidated with the UV-Vis spectra. The results obtained with the assumption of direct transitions matched those that would have been obtained using, for example, the cut-off wavelength method very well. More conservative results were obtained using the ASF method, which are given here. LDHs have been described to have a complex band structure and consist of multiple absorption bands [16]. Such a structure was visible for MgAl-LDH, albeit the total absorbance not being very high for this material. The absorption bands were very small and almost invisible in comparison to the other materials as shown in Figure 7. MgAl-LDH displayed three small absorption bands with bandgaps of 2.32 eV, 3.84 eV, and 4.43 eV. MgFeAl-LDH had a bandgap of 1.88 eV. Upon modification with Coumarin 153, the UV-Vis absorption of the MgAl-LDH+dye complex was enhanced, leading to a modified material with a bandgap of 2.14 eV. The TiO_2_ used for comparative purposes consisted of 89 vol% anatase and 11 vol% rutile. Anatase is an indirect bandgap semiconductor and rutile a direct bandgap semiconductor [24,39]. The combination of these two leads to a lowering in the bandgap of the overall material [40]. For the bandgap determination, because the material consisted of 89 vol% anatase, indirect transitions were used. Through the ASF method, the bandgap of TiO_2_ and the TiO_2_+dye complex were determined to be 2.87 eV and 2.34 eV respectively, about 0.5 eV to 1 eV higher than that of the MgFeAl-LDH.

Finally, the photovoltaic potential of the LDHSCs was studied using a current-voltage (I-V) tester and a solar simulator. The values obtained from the I-V tester under solar simulation are shown in Table 2. The IV graphs obtained of the prepared cells are shown in Figure 7.

Under the simulated AM 1.5 illumination, SCN4 showed an open circuit voltage (V_OC_) of 726 mV. In comparison, SCN2 and SCN3 only achieved an open circuit voltage of 69 mV and 81 mV, respectively. SCN1 showed no functionality, the results were thus excluded from the table. Without the use of Coumarin 153, MgFeAl (SCN4) surpassed the open circuit voltages of the other cells by multifold and achieved a power conversion efficiency of 1.56%. SCN1, SCN2, and SCN3 showed no or very low efficiencies of 0%, 0.0009%, and 0.0012%, respectively.

The MgFeAl-LDH-based cell without dye-sensitization thus significantly outperformed the other cells tested, which can be correlated to its superior UV-Vis absorption behavior (as shown in Figure 5) both in intensity of the absorption band and increase in the absorption range, extending far into the visible. Typically, semiconductors are calcined onto the electrode to reduce contact resistance between the two materials. It is believed that the omission of this step and lower overall UV-Vis absorption led to the low efficiency of the TiO_2_-based cells. The MgAl-LDH-based cell without dye-sensitization showed no functionality, which is believed to have resulted from the minor absorption capacity of the material in comparison to MgFeAl-LDH. The material had three small absorption bands as identified in Figure 6a. Only one of them was well defined, albeit a small band, at 300 nm and two of them were broader and less well-defined. The dye-sensitized MgAl-LDH material showed a better efficiency, although not better than the dye-sensitized TiO_2,_ which had a lower overall UV-Vis absorption range and intensity. This is believed to have resulted from a mismatch between the dye and MgAl-LDH as can be observed in the UV-Vis spectra in Figure 5, where the absorption band of the dye and small absorption band of the MgAl-LDH do not overlap significantly, only the ill-defined bands show some overlap with the dye. Efficient electron injection into the conduction band of the LDH would thus likely have been hindered. For TiO_2_, an appropriate overlap of the absorption band of the dye and the semiconductor can be observed in Figure 5, which is mirrored in the increased performance of this material. This material was also of smaller particle size, thus facilitating better contact with the electrode.

The results from the I-V testing of the cells show that the replacement of the TiO_2_+dye complex in DSSCs with a UV-Vis photoabsorbing LDH can achieve conversion efficiencies of up to 1.56%. Some of the main problems in DSSCs are the cost and environmental concerns associated with the use of dyes as photosensitizers and electrolyte leakage [13]. The dyes are typically added to increase absorbance efficiency of the semiconductor in the Vis-light region. Use of an UV-Vis absorbing LDH showed that it is possible to circumvent the necessity of dye-sensitization by the LDH acting as an effective simultaneous photoabsorber and charge separator—with far greater absorbance than the TiO_2_+dye complex—that is able to induce an electron flow. It is believed that the Fe incorporated into the LDH facilitated this flow of electrons by acting as a redox couple.

## 4. Conclusions

With the use of UV-Vis photoabsorbing LDHs it was possible to replace the dye+semiconductor complex used in DSSCs, giving an alternative to dye-sensitization. The resulting dye-free LDHSC comprised of a simple design that achieved a 1.56% efficiency. Even with its simple set-up, it surpassed the efficiency of many other, much more complex, DSSCs containing MMOs/LDOs derived from LDHs and made the use of expensive dye redundant. Using LDHs instead of dye+semiconductor complexes could open up research into low-cost photovoltaic devices.

As with typical DSSCs, there remain many different options to increase the efficiency of LDHSCs. It remains to be determined how stable these separated charges are or through which mechanism they are transferred to the respective electrodes and what the materials’ stability through interaction of the electrolyte would be. One of the key elements to this performance is the Fe-substitution in LDH. This phenomenon could be further explored by using other Fe concentrations. Better efficiency is also expected through decreased contact resistance in the cell. Further work on the application of photoactive LDH in this way and also in other cell design concepts is in progress and improvements in the design and efficiency are expected.

## Figures and Tables

**Table 1:** Layered double hydroxides (LDH)-based and TiO_2_-based solar cell compositions and structures.

ID	SCN1	SCN2	SCN3	SCN4
**Setup**	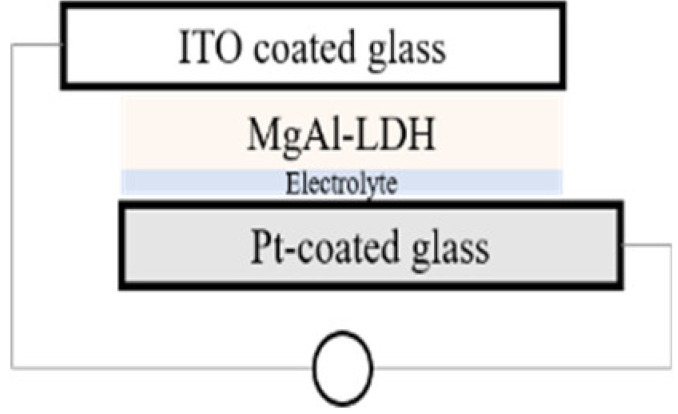	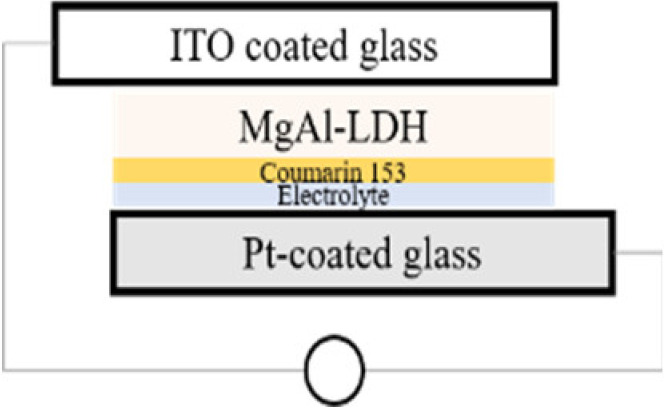	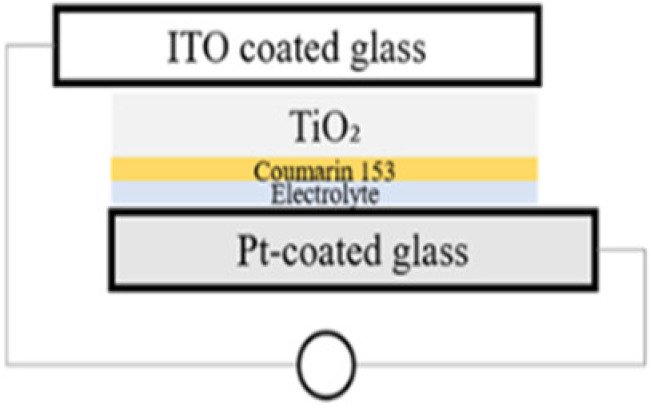	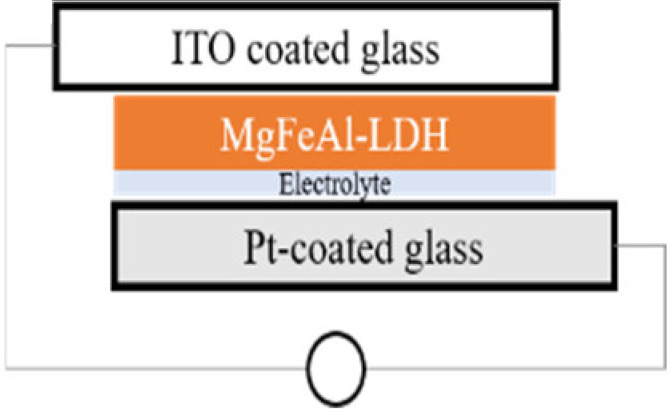
**Detail**	MgAl (2.10 mg) + electrolyte (0.0192 mL)	MgAl (2.15 mg) + dye (Coumarin 153) (0.0096 mL) + electrolyte (0.0192 mL)	TiO_2_ (2.15 mg) + dye (Coumarin 153) (0.0096 mL) + electrolyte (0.0192 mL)	MgFeAl (2.21 mg) + electrolyte (0.0192 mL)

**Table 2:** Photovoltaic performance of the layered double hydroxide solar cells (LDHSCs) tested. SCN2 (MgAl-LDH+dye), SCN3 (TiO_2_+dye), and SCN4 (MgFeAl-LDH). The fill factor (FF), open circuit voltage (V_OC_), short circuit current (I_SC_), and efficiency (η) are shown.

	FF	V_OC_ (mV)	I_SC_ (mA)	η (%)
SCN2	0.945	69	0.00182	0.0009
SCN3	0.941	81	0.002	0.0012
SCN4	0.727	726	0.371	1.56
